# BMD in peritoneal dialysis: what BRAZPD can help us understand?

**DOI:** 10.1590/2175-8239-JBN-2021-E003

**Published:** 2021-05-28

**Authors:** Melani Custodio

**Affiliations:** 1 Universidade de São Paulo, Laboratório de Fisiopatologia Renal, São Paulo, SP, Brasil.

The Brazilian Study of Peritoneal Dialysis II (BRAZPD II) was a milestone in clinical research, as it was the first large cohort in Latin America to show characteristics of patients on peritoneal dialysis (PD), clinical practices, techniques and their correlations with clinical results. One hundred and twenty-two clinics from different parts of the country participated in the study, with 9,905 patients, from December 2004 to January 2011.

The data collected from this cohort gave rise to other studies, resulting in 33 published papers and 627 citations, which shows the impact and importance of the BRAZPD in the nephrological community.

Studies on mineral and bone metabolism disorder (BMD) in PD, most of which were carried out with a small number of patients, date from 1982. These studies were largely associated and addressed the pathophysiology of renal osteodystrophy (RO), the concentration of calcium (Ca) in the dialysate and the use of Ca-based phosphorus chelators, among others. In the past, the possibilities of controlling biochemical changes in PD were limited, boiling down to the use of calcium carbonate (CaCO3) to control hyperphosphatemia and to manage the Ca concentration in the dialysis bath to treat hypercalcemia. Subsequently, in the 1990s, calcitriol and sevelamer were added for the treatment of secondary hyperparathyroidism (HPTS) and, in 2016, cinacalcet and paricalcitol.

Recently, the BJN published two more studies from the BRAZPD:

1. **"High prevalence of biochemical disturbances of chronic kidney disease mineral and bone disorders (CKD-MBD) in a nation-wide peritoneal dialysis cohort: are guideline goals too hard to achieve?"^1^**

This paper, with a patient follow-up of 12 months, made it possible to show the prevalence of metabolic changes in PD patients, which were similar to those of patients on hemodialysis (HD). Regarding the percentage of patients who reached the goals established by the KDOQI, the current guideline at the time, there was a small variation between the beginning and the end of the study. The results showed that 50% of the patients reached the goals for Ca and P, and only 26% of the patients reached the goal for PTH, despite the use of CaCO3, sevelamer and calcitriol. Another relevant fact was the increase in the number of patients with hypercalcemia, probably due to the use of a dialysate with a Ca 3.5 mEq/L concentration, associated with the use of Ca-based phosphorus chelators. Considering the duration of data collection for this study, there are some factors that probably hindered the achievement of better goals: dosage and replacement of 25(OH) vitamin D, which was done in only 25% of the patients, little availability of drugs for the treatment of BMD, aggravated by the excess of bureaucracy for its distribution.

This study points out that, just like HD patients, PD patients have important BMD and need more effective control, aiming to reduce the risk of mortality and improve quality of life.

2. **"Cardiovascular mortality in peritoneal dialysis: the impact of mineral disorders"^2^**

The BRAZPD enabled the analysis of BMD in 65-70% of PD patients from all over Brazil, with a sample of 4,424 incident patients. It is worth mentioning that, for this study, 2 models were created to assess the association of Ca, P, PTH and cardiovascular mortality, according to the ideal ranges recommended by the KDOQI and the KDIGO. The results showed that high P and low PTH, for both guidelines, were associated with cardiovascular mortality. Another interesting finding (data not shown) was the incidence of bone diseases: low remodeling disease (PTH <150 pg/mL) affected 30% of patients, considering the two guidelines; and high remodeling disease occurred in 39% of patients, according to the KDOQI, and 16% of patients, according to the KDIGO. These results showed the diversity of BMD in PD, and the incidence of adynamic disease is lower than the assumption, with an important number of patients with high remodeling disease.

In the last two decades, numerous studies have shown the association between BMD and cardiovascular mortality, responsible for 50% of deaths in dialysis patients. Most of these studies were performed on patients in the pre-dialysis phase or on hemodialysis (HD). The findings of PD patients are not always coincident with the others, except for the results of phosphorus, which is associated with mortality, both in very low and very high concentrations.

These two papers mentioned contributed to the knowledge and management of BMD in PD patients until 2011. Today, as in HD patients, we need to increase our knowledge of bone mineral disorders and their implications in PD patients, such as vascular calcification, the risk of fractures, the incidence and treatment of osteoporosis, among others. Thus, we can really offer patients an individualized treatment, decreasing cardiovascular mortality, the risk of fractures and other complications.

Some resources are suggested in the treatment of BMD in PD patients, such as:

1. Limit the use of Ca-based phosphorus chelators, avoiding a positive Ca balance and complications such as vascular calcification and calciphylaxis^3^;
2. Adjust the Ca concentration in the dialysis bath, using a 2.5% mEq/L solution for patients with PTH <150 pg/mL^4^;
3. Avoid PTH levels <150 pg/mL and P below the recommended level, which are associated with a higher risk of peritonitis and mortality^5^^,^ 6;
4. Consider a higher risk of all-cause mortality with alkaline phosphatase (FA) ≧ 100 IU/L and PTH <100 pg/mL^7^;
5. Consider replacement of 25(OH) vitamin D, when necessary^8^;
6. Assess the risk of fractures, which contributes to increased mortality, through bone densitometry (DXA)^9^.

In conclusion, we need to look at PD patients with greater care, so that more goals for the control of BMD are achieved, resulting in longer survival and better quality of life for these patients.

## References

[1] Weissheimer R, Bucharles SGE, Truyts CAM, Jorgetti V, Figueiredo AE, Barrett P (2021). High prevalence of biochemical disturbances of chronic kidney
disease - mineral and bone disorders (CKD-MBD) in a nation-wide peritoneal
dialysis cohort: are guideline goals too hard to achieve?. Braz J Nephrol.

[2] Truyts C, Custodio M, Pecoit-Filho R, Moraes TP, Jorgetti V (2021). Cardiovascular mortality in peritoneal dialysis: the impact of
mineral disorders. Braz J Nephrol.

[3] Kidney Disease: Improving Global Outcomes (KDIGO) (2017). KDIGO 2017 Clinical practice guideline update for the diagnosis,
evaluation, prevention, and treatment of chronic kidney disease-mineral and
bone disorder (CKD-MBD). Kidney Int Suppl.

[4] Moraes TP, Bucharles SGE, Ribeiro SC, Frumento R, Riella MC, Pecoits-Filho R (2010). Low-calcium peritoneal dialysis solution is effective in bringing
PTH levels to the range recommended by current guidelines in patients with
PTH levels < 150 pg/dL. Braz J Nefrol.

[5] Yang Y, Da J, Jiang Y, Yuan J, Zha Y (2021). Low serum parathyroid hormone is a risk factor for peritonitis
episodes incident peritoneal dialysis patients: a retrospective
study. BMC Nephrol.

[6] Hong YA, Kim JH, Kim YK, Chang YK, Park CW, Kim SY (2020). Low parathyroid hormone level predicts infection-related
mortality in incident dialysis patients: a prospective cohort
study. Korean J Intern Med.

[7] Chen Z, Zhang X, Han F, Xie X, Hua Z, Huang X (2021). High alkaline phosphatase and low intact parathyroid hormone
associate with worse clinical outcome in peritoneal dialysis
patients. Perit Dial Int.

[8] Pi HC, Ren YP, Wang Q, Xu R, Dong J (2015). Serum 25-hydroxyvitamin D level could predict the risk for
peritoneal dialysis-associated peritonitis. Perit Dial Int.

[9] Iseri C, Carrero JJ, Evans M, Felländer-Tsai L, Berg H, Runesson B (2020). Major fractures after initiation of dialysis: incidence,
predictors and association with mortality. Bone.

